# Bis(μ-diisopropyl­hydoxylaminato)-κ^2^ *O*:*N*;κ^2^ *O*:*O*-bis­[(diisopropyl­hydoxylaminato-κ*O*)beryllium]

**DOI:** 10.1107/S1600536812045655

**Published:** 2012-11-10

**Authors:** Raphael Johann Friedrich Berger, Surajit Jana, Uwe Monkowius, Norbert Werner Mitzel

**Affiliations:** a Fachbereich für Materialwissenschaften und Physik, Paris-Lodron Universität Salzburg, 5020 Salzburg, Austria; b Department of Chemistry, Asansol Girls’ College, Asansol 713 304, West Bengal, India; c Institut für Anorganische Chemie, Johannes-Kepler-Universität Linz, Altenbergerstrasse 69, 4040 Linz, Austria; d Lehrstuhl für Anorganische Chemie und Strukturchemie, Universität Bielefeld, 33615 Bielefeld, Germany

## Abstract

The title compound, [Be_2_(C_6_H_14_NO)_4_], was prepared from a solution of BeCl_2_ in diethyl ether and two equivalents of *O*-lithia­ted *N*,*N*-diisopropyl­hydoxyl­amine. The mol­ecular structure is composed of a dinuclear unit forming a central five-membered planar Be—O—Be—O—N ring (sum of inter­nal angles = 540.0°; r.m.s. deviation from planarity = 0.0087 Å). Both Be atoms show the unusual coordination number of three, with one Be atom coordinated by three O atoms and the other by two O atoms and one N atom, both in distorted trigonal–planar environments. The Be—O distances are in the range 1.493 (5)–1.600 (5) Å and the Be—N distance is 1.741 (5) Å.

## Related literature

For general background to metal compounds containing hydroxyl­amine ligands, see: Ullrich (2007 ▶). For further information on beryllium coordination compounds, see: Berger, Hartmann *et al.* (2001 ▶); Berger, Schmidt *et al.* (2001 ▶); Dressel *et al.* (2003 ▶); Berger *et al.* (2011 ▶); for information about coordination compounds containing Be—N bonds, see: Dressel *et al.* (2003 ▶); Neumüller & Dehnicke (2010 ▶).
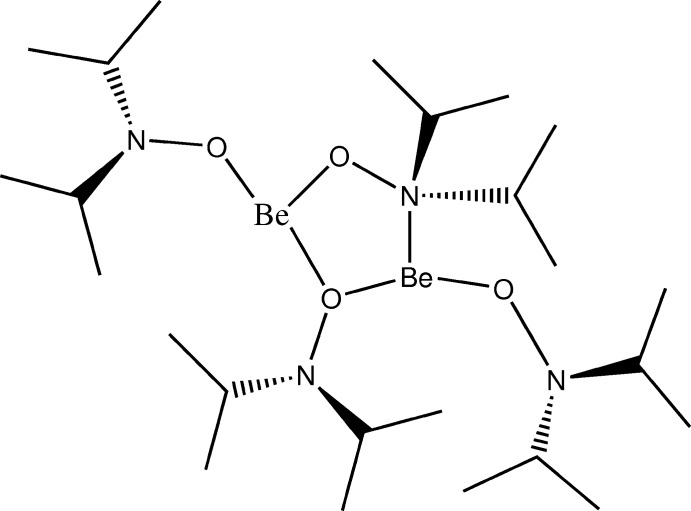


## Experimental

### 

#### Crystal data

[Be_2_(C_6_H_14_NO)_4_]
*M* *_r_* = 482.75
Triclinic, 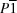

*a* = 8.2156 (17) Å
*b* = 13.248 (3) Å
*c* = 14.317 (3) Å
α = 98.82 (2)°
β = 97.42 (2)°
γ = 102.42 (2)°
*V* = 1482.5 (5) Å^3^
*Z* = 2
Mo *K*α radiation
μ = 0.07 mm^−1^
*T* = 110 K
0.20 × 0.10 × 0.05 mm

#### Data collection

Stoe IPDS diffractometer
20278 measured reflections
5220 independent reflections
2359 reflections with *I* > 2σ(*I*)
*R*_int_ = 0.162

#### Refinement

*R*[*F* ^2^ > 2σ(*F* ^2^)] = 0.061
*wR*(*F* ^2^) = 0.169
*S* = 0.98
5220 reflections
323 parameters
H-atom parameters constrained
Δρ_max_ = 0.17 e Å^−3^
Δρ_min_ = −0.22 e Å^−3^

### 

Data collection: *X-AREA* (Stoe & Cie, 2012 ▶); cell refinement: *X-AREA*; data reduction: *X-RED32* (Stoe & Cie, 2012 ▶); program(s) used to solve structure: *SHELXS97* (Sheldrick, 2008 ▶); program(s) used to refine structure: *SHELXL97* (Sheldrick, 2008 ▶); molecular graphics: *PLATON* (Spek, 2009 ▶); software used to prepare material for publication: *publCIF* (Westrip, 2010 ▶).

## Supplementary Material

Crystal structure: contains datablock(s) I, global. DOI: 10.1107/S1600536812045655/wm2695sup1.cif

Structure factors: contains datablock(s) I. DOI: 10.1107/S1600536812045655/wm2695Isup2.hkl

Additional supplementary materials:  crystallographic information; 3D view; checkCIF report

## Figures and Tables

**Fig. 1.**
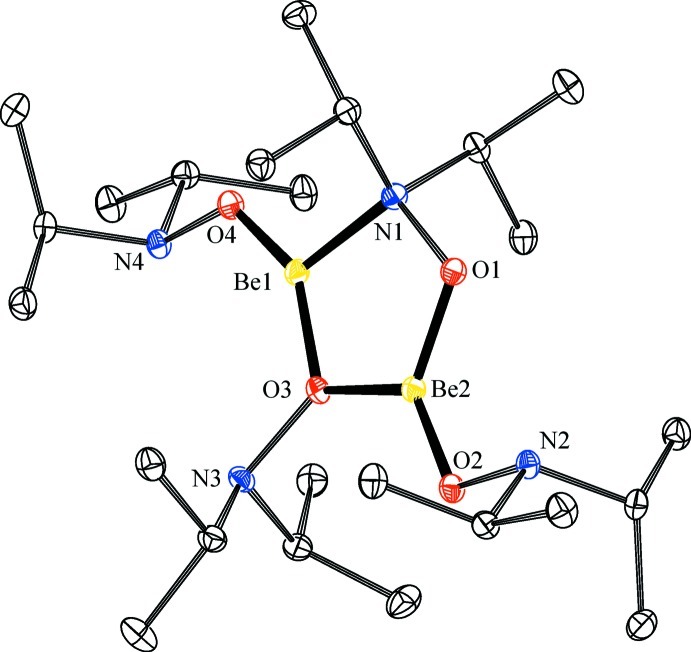
The dinuclear molecular unit of the title compound. Atoms are displayed with displacement ellipsoids at the 10% probability level. Hydrogen atoms have been omitted for clarity.

## supplementary crystallographic information

### Comment

A large number of coordination compounds of organo-substituted hydoxylamines with electropositive elements *M* is known up to date (Ullrich, 2007). Depending on the tendency of *M* to form ionic, covalent, or coordinative bonds to O and N atoms of the hydroxyl amine moiety, and also depending on the valency and the size of *M*, a comparably large variety of structure motifs and compositions is found. *N*,*N*-diisopropylhydoxylamine is probably the sterically most hindered hydroxylamine which is accessible. In continuation of our studies on Be coordination compounds (Berger, Hartmann *et al.*, 2001; Berger *et al.*, 2011; Berger, Schmidt *et al.*, 2001; Dressel *et al.*, 2003; Neumüller & Dehnicke, 2010), it was of special interest to investigate the reaction products formed from Be^2+^ and deprotonated *N*,*N*-diisopopylhydoxylamine.

In the dinuclear unit of the title compound, Be_2_(C_3_H_7_NO)_4_, the Be^2+^ cations are bonded to O atoms and one N atom of the hydroxylamine moieties. The mononuclear subunit Be(ON*^i^*Pr_2_)_2_ is connected *via* an additional Be—O bond to an identical subunit. In addition, a Be—N bond to one of the four hydroxylamine moieties is observed. In this way, each of the two Be^2+^ cations attains a threefold coordination. (Fig. 1) Such a coordination number is rarely found in Be-containing compounds and usually only in sterically demanding environments. Another striking feature of the molecular arrangement of the title compound is the planarity of the central Be—O—Be—O—N five-membered ring. This is underlined by the sum of 540.0° of the internal angles in the ring and a r.m.s. deviation from planarity of only 0.0087 Å. Despite the planarity, no crystallographically imposed symmetry is found in the molecular unit of Be_2_(C_3_H_7_NO)_4_.

### Experimental

A suspension of 1.282 g (4 mmol) Li(ON*^i^*Pr_2_) in 15 ml of dry diethyl ether was slowly dropped to a solution of 159 mg BeCl_2_ (2 mmol) in 5 ml diethyl ether. The reaction mixture was stirred for 6 h at room temperature and filtered using a syringe equipped with a Whatmann glass filter. The clear solution was reduced *in vacuo* to about 15 ml and stored at 243 K for two weeks. Clear colorless block-shaped crystals were collected from the solution under an argon atmosphere. The material is extremely water- and moist-sensitive and must be handled under inert gas.

### Refinement

All H atoms were treated as riding with C—H distances of 0.97 (C—H), 0.98 (CH_3_), and *U*_iso_(H) = 1.2 *U*_eq_(C) [1.5 *U*_eq_ for methyl hydrogen atoms]. Riding methyl hydrogen atoms were allowed to rotate freely during refinement.

### Crystal data

**Table:**

[Be_2_(C_6_H_14_NO)_4_]	*Z* = 2
*M**_r_* = 482.75	*F*(000) = 536
Triclinic, *P*1	*D*_x_ = 1.081 Mg m^−^^3^
Hall symbol: -P 1	Mo *K*α radiation, λ = 0.71073 Å
*a* = 8.2156 (17) Å	Cell parameters from 5000 reflections
*b* = 13.248 (3) Å	θ = 4.3–25.2°
*c* = 14.317 (3) Å	µ = 0.07 mm^−^^1^
α = 98.82 (2)°	*T* = 110 K
β = 97.42 (2)°	Block, colourless
γ = 102.42 (2)°	0.20 × 0.10 × 0.05 mm
*V* = 1482.5 (5) Å^3^	

### Data collection

**Table:**

Stoe IPDS diffractometer	2359 reflections with *I* > 2σ(*I*)
Radiation source: sealed tube	*R*_int_ = 0.162
Graphite monochromator	θ_max_ = 25.8°, θ_min_ = 4.2°
rotation scans	*h* = −10→9
20278 measured reflections	*k* = −16→16
5220 independent reflections	*l* = −17→17

### Refinement

**Table:**

Refinement on *F*^2^	Primary atom site location: structure-invariant direct methods
Least-squares matrix: full	Secondary atom site location: difference Fourier map
*R*[*F*^2^ > 2σ(*F*^2^)] = 0.061	Hydrogen site location: inferred from neighbouring sites
*wR*(*F*^2^) = 0.169	H-atom parameters constrained
*S* = 0.98	*w* = 1/[σ^2^(*F*_o_^2^) + (0.0603*P*)^2^] where *P* = (*F*_o_^2^ + 2*F*_c_^2^)/3
5220 reflections	(Δ/σ)_max_ < 0.001
323 parameters	Δρ_max_ = 0.17 e Å^−^^3^
0 restraints	Δρ_min_ = −0.22 e Å^−^^3^
0 constraints	

### Special details

**Table:**

Geometry. All e.s.d.'s (except the e.s.d. in the dihedral angle between two l.s. planes) are estimated using the full covariance matrix. The cell e.s.d.'s are taken into account individually in the estimation of e.s.d.'s in distances, angles and torsion angles; correlations between e.s.d.'s in cell parameters are only used when they are defined by crystal symmetry. An approximate (isotropic) treatment of cell e.s.d.'s is used for estimating e.s.d.'s involving l.s. planes.
Refinement. Refinement of *F*^2^ against ALL reflections. The weighted *R*-factor *wR* and goodness of fit *S* are based on *F*^2^, conventional *R*-factors *R* are based on *F*, with *F* set to zero for negative *F*^2^. The threshold expression of *F*^2^ > σ(*F*^2^) is used only for calculating *R*-factors(gt) *etc*. and is not relevant to the choice of reflections for refinement. *R*-factors based on *F*^2^ are statistically about twice as large as those based on *F*, and *R*- factors based on ALL data will be even larger.

### Fractional atomic coordinates and isotropic or equivalent isotropic displacement parameters (Å^2^)

**Table:**

	*x*	*y*	*z*	*U*_iso_*/*U*_eq_	
O1	0.0493 (3)	0.11429 (16)	0.64521 (17)	0.0425 (6)	
N1	0.1242 (3)	0.1998 (2)	0.6006 (2)	0.0406 (7)	
Be1	0.2326 (6)	0.3041 (3)	0.6921 (3)	0.0413 (11)	
O2	0.0366 (3)	0.07170 (16)	0.82450 (17)	0.0454 (6)	
N2	−0.0419 (3)	−0.03765 (19)	0.7809 (2)	0.0415 (7)	
Be2	0.0876 (5)	0.1400 (3)	0.7543 (3)	0.0399 (11)	
O3	0.1982 (3)	0.25866 (16)	0.78337 (17)	0.0415 (6)	
N3	0.2877 (4)	0.3196 (2)	0.8786 (2)	0.0437 (7)	
O4	0.3156 (3)	0.39645 (16)	0.65618 (18)	0.0449 (6)	
N4	0.3860 (3)	0.50261 (19)	0.7113 (2)	0.0408 (7)	
C11	0.2354 (4)	0.1547 (3)	0.5371 (3)	0.0446 (9)	
H11	0.1673	0.0866	0.4967	0.054*	
C12	0.3817 (5)	0.1326 (3)	0.6004 (3)	0.0560 (10)	
H12A	0.4490	0.0977	0.5603	0.084*	
H12B	0.3376	0.0869	0.6437	0.084*	
H12C	0.4529	0.1991	0.6382	0.084*	
C13	0.3003 (6)	0.2276 (3)	0.4717 (3)	0.0601 (11)	
H13A	0.2059	0.2320	0.4244	0.090*	
H13B	0.3835	0.2003	0.4386	0.090*	
H13C	0.3537	0.2977	0.5097	0.090*	
C14	−0.0187 (4)	0.2287 (3)	0.5431 (3)	0.0468 (9)	
H14	0.0312	0.2870	0.5109	0.056*	
C15	−0.1278 (5)	0.2706 (3)	0.6101 (3)	0.0577 (11)	
H15A	−0.1862	0.2133	0.6386	0.087*	
H15B	−0.2114	0.2990	0.5739	0.087*	
H15C	−0.0563	0.3264	0.6610	0.087*	
C16	−0.1257 (5)	0.1398 (3)	0.4662 (3)	0.0623 (12)	
H16A	−0.0545	0.1152	0.4222	0.094*	
H16B	−0.2144	0.1648	0.4305	0.094*	
H16C	−0.1778	0.0818	0.4959	0.094*	
C21	0.0464 (5)	−0.1004 (3)	0.8373 (3)	0.0476 (9)	
H21	0.0360	−0.0819	0.9064	0.057*	
C22	−0.0306 (5)	−0.2169 (3)	0.8010 (3)	0.0647 (12)	
H22A	−0.1447	−0.2352	0.8167	0.097*	
H22B	0.0397	−0.2577	0.8316	0.097*	
H22C	−0.0365	−0.2330	0.7314	0.097*	
C23	0.2317 (5)	−0.0756 (3)	0.8273 (3)	0.0619 (11)	
H23A	0.2415	−0.0873	0.7592	0.093*	
H23B	0.2889	−0.1215	0.8598	0.093*	
H23C	0.2844	−0.0019	0.8565	0.093*	
C24	−0.2226 (4)	−0.0542 (3)	0.7891 (3)	0.0484 (10)	
H24	−0.2763	−0.1304	0.7633	0.058*	
C25	−0.3057 (5)	0.0085 (3)	0.7246 (3)	0.0576 (11)	
H25A	−0.2711	0.0834	0.7537	0.086*	
H25B	−0.4289	−0.0157	0.7170	0.086*	
H25C	−0.2705	−0.0019	0.6617	0.086*	
C26	−0.2600 (5)	−0.0305 (3)	0.8900 (3)	0.0624 (11)	
H26A	−0.2184	−0.0779	0.9283	0.094*	
H26B	−0.3823	−0.0409	0.8878	0.094*	
H26C	−0.2034	0.0425	0.9193	0.094*	
C31	0.1634 (5)	0.3670 (3)	0.9256 (3)	0.0508 (10)	
H31	0.2247	0.4063	0.9902	0.061*	
C32	0.1153 (5)	0.4483 (3)	0.8714 (3)	0.0613 (11)	
H32A	0.2171	0.4911	0.8552	0.092*	
H32B	0.0613	0.4936	0.9114	0.092*	
H32C	0.0364	0.4129	0.8124	0.092*	
C33	0.0087 (6)	0.2913 (3)	0.9430 (4)	0.0710 (13)	
H33A	−0.0626	0.2561	0.8815	0.106*	
H33B	−0.0554	0.3302	0.9821	0.106*	
H33C	0.0441	0.2386	0.9768	0.106*	
C34	0.3584 (5)	0.2466 (3)	0.9307 (3)	0.0508 (10)	
H34	0.2658	0.1855	0.9350	0.061*	
C35	0.4895 (5)	0.2079 (3)	0.8798 (3)	0.0597 (11)	
H35A	0.4369	0.1708	0.8147	0.090*	
H35B	0.5359	0.1598	0.9151	0.090*	
H35C	0.5806	0.2679	0.8765	0.090*	
C36	0.4425 (6)	0.3052 (4)	1.0308 (3)	0.0769 (14)	
H36A	0.5129	0.3737	1.0267	0.115*	
H36B	0.5132	0.2641	1.0606	0.115*	
H36C	0.3555	0.3158	1.0696	0.115*	
C41	0.3229 (4)	0.5723 (3)	0.6515 (3)	0.0467 (9)	
H41	0.3582	0.5599	0.5872	0.056*	
C42	0.1321 (5)	0.5490 (3)	0.6387 (3)	0.0613 (11)	
H42A	0.0842	0.4764	0.6048	0.092*	
H42B	0.0914	0.5974	0.6013	0.092*	
H42C	0.0969	0.5581	0.7017	0.092*	
C43	0.3965 (5)	0.6860 (3)	0.6994 (3)	0.0624 (12)	
H43A	0.3752	0.6961	0.7656	0.094*	
H43B	0.3433	0.7316	0.6642	0.094*	
H43C	0.5186	0.7039	0.6994	0.094*	
C44	0.5723 (4)	0.5197 (3)	0.7237 (3)	0.0470 (9)	
H44	0.6210	0.5937	0.7584	0.056*	
C45	0.6278 (5)	0.4488 (3)	0.7884 (3)	0.0603 (11)	
H45A	0.5682	0.4513	0.8434	0.090*	
H45B	0.7500	0.4728	0.8111	0.090*	
H45C	0.6013	0.3764	0.7526	0.090*	
C46	0.6461 (5)	0.5079 (3)	0.6317 (3)	0.0604 (11)	
H46A	0.5945	0.4380	0.5929	0.091*	
H46B	0.7686	0.5165	0.6475	0.091*	
H46C	0.6230	0.5618	0.5955	0.091*	

### Atomic displacement parameters (Å^2^)

**Table:**

	*U*^11^	*U*^22^	*U*^33^	*U*^12^	*U*^13^	*U*^23^
O1	0.0456 (13)	0.0334 (12)	0.0449 (16)	0.0015 (10)	0.0067 (11)	0.0084 (10)
N1	0.0435 (15)	0.0342 (14)	0.0440 (19)	0.0069 (12)	0.0066 (13)	0.0109 (12)
Be1	0.040 (2)	0.037 (2)	0.042 (3)	0.0016 (19)	0.009 (2)	0.0029 (19)
O2	0.0464 (14)	0.0352 (12)	0.0473 (16)	0.0003 (10)	0.0035 (11)	0.0022 (10)
N2	0.0387 (15)	0.0319 (14)	0.049 (2)	0.0029 (12)	0.0026 (14)	0.0058 (13)
Be2	0.038 (2)	0.038 (2)	0.041 (3)	0.0036 (18)	0.005 (2)	0.0078 (19)
O3	0.0405 (12)	0.0366 (12)	0.0399 (15)	0.0030 (10)	−0.0006 (10)	−0.0006 (10)
N3	0.0471 (17)	0.0424 (16)	0.0347 (18)	0.0055 (13)	−0.0002 (14)	−0.0011 (12)
O4	0.0483 (13)	0.0320 (12)	0.0477 (16)	0.0022 (10)	0.0048 (11)	0.0003 (10)
N4	0.0420 (15)	0.0273 (14)	0.0484 (19)	0.0031 (11)	0.0020 (13)	0.0050 (12)
C11	0.049 (2)	0.0362 (18)	0.045 (2)	0.0063 (15)	0.0083 (17)	0.0022 (15)
C12	0.052 (2)	0.053 (2)	0.060 (3)	0.0120 (18)	0.0070 (19)	0.0014 (18)
C13	0.075 (3)	0.050 (2)	0.055 (3)	0.008 (2)	0.021 (2)	0.0089 (18)
C14	0.048 (2)	0.0366 (18)	0.051 (2)	0.0068 (15)	−0.0053 (18)	0.0097 (16)
C15	0.047 (2)	0.054 (2)	0.071 (3)	0.0150 (18)	0.005 (2)	0.010 (2)
C16	0.065 (3)	0.051 (2)	0.062 (3)	0.0111 (19)	−0.012 (2)	0.0035 (19)
C21	0.051 (2)	0.0422 (19)	0.047 (2)	0.0076 (16)	0.0034 (18)	0.0115 (16)
C22	0.069 (3)	0.045 (2)	0.075 (3)	0.0069 (19)	0.003 (2)	0.012 (2)
C23	0.050 (2)	0.059 (2)	0.076 (3)	0.0136 (19)	0.008 (2)	0.010 (2)
C24	0.0353 (19)	0.047 (2)	0.058 (3)	0.0014 (15)	0.0062 (18)	0.0097 (17)
C25	0.048 (2)	0.062 (2)	0.064 (3)	0.0139 (18)	0.004 (2)	0.015 (2)
C26	0.054 (2)	0.072 (3)	0.066 (3)	0.012 (2)	0.020 (2)	0.020 (2)
C31	0.055 (2)	0.049 (2)	0.043 (2)	0.0100 (17)	0.0112 (18)	−0.0044 (17)
C32	0.057 (2)	0.057 (2)	0.068 (3)	0.0160 (19)	0.013 (2)	0.003 (2)
C33	0.073 (3)	0.063 (3)	0.079 (4)	0.014 (2)	0.038 (3)	0.004 (2)
C34	0.055 (2)	0.052 (2)	0.042 (2)	0.0074 (18)	0.0027 (18)	0.0120 (17)
C35	0.055 (2)	0.059 (2)	0.065 (3)	0.0181 (19)	−0.001 (2)	0.016 (2)
C36	0.090 (3)	0.074 (3)	0.054 (3)	0.016 (2)	−0.018 (2)	0.001 (2)
C41	0.050 (2)	0.0396 (19)	0.050 (2)	0.0095 (16)	0.0088 (18)	0.0101 (16)
C42	0.048 (2)	0.056 (2)	0.078 (3)	0.0118 (18)	−0.002 (2)	0.018 (2)
C43	0.064 (3)	0.038 (2)	0.078 (3)	0.0074 (18)	0.000 (2)	0.0071 (19)
C44	0.0338 (18)	0.0428 (19)	0.059 (3)	0.0017 (15)	0.0042 (17)	0.0068 (17)
C45	0.045 (2)	0.067 (3)	0.066 (3)	0.0125 (19)	0.002 (2)	0.015 (2)
C46	0.050 (2)	0.060 (2)	0.075 (3)	0.0095 (19)	0.021 (2)	0.018 (2)

### Geometric parameters (Å, º)

**Table:**

O1—N1	1.447 (3)	C24—C26	1.516 (6)
O1—Be2	1.522 (5)	C24—C25	1.525 (5)
N1—C14	1.498 (4)	C24—H24	1.0000
N1—C11	1.517 (5)	C25—H25A	0.9800
N1—Be1	1.741 (5)	C25—H25B	0.9800
Be1—O4	1.467 (5)	C25—H25C	0.9800
Be1—O3	1.554 (5)	C26—H26A	0.9800
Be1—Be2	2.587 (6)	C26—H26B	0.9800
O2—N2	1.457 (3)	C26—H26C	0.9800
O2—Be2	1.493 (5)	C31—C32	1.509 (6)
N2—C24	1.476 (4)	C31—C33	1.514 (5)
N2—C21	1.478 (4)	C31—H31	1.0000
Be2—O3	1.600 (5)	C32—H32A	0.9800
O3—N3	1.483 (3)	C32—H32B	0.9800
N3—C34	1.476 (5)	C32—H32C	0.9800
N3—C31	1.488 (5)	C33—H33A	0.9800
O4—N4	1.459 (3)	C33—H33B	0.9800
N4—C41	1.479 (4)	C33—H33C	0.9800
N4—C44	1.481 (4)	C34—C35	1.510 (6)
C11—C13	1.509 (5)	C34—C36	1.518 (5)
C11—C12	1.517 (5)	C34—H34	1.0000
C11—H11	1.0000	C35—H35A	0.9800
C12—H12A	0.9800	C35—H35B	0.9800
C12—H12B	0.9800	C35—H35C	0.9800
C12—H12C	0.9800	C36—H36A	0.9800
C13—H13A	0.9800	C36—H36B	0.9800
C13—H13B	0.9800	C36—H36C	0.9800
C13—H13C	0.9800	C41—C42	1.512 (5)
C14—C16	1.511 (5)	C41—C43	1.514 (5)
C14—C15	1.517 (6)	C41—H41	1.0000
C14—H14	1.0000	C42—H42A	0.9800
C15—H15A	0.9800	C42—H42B	0.9800
C15—H15B	0.9800	C42—H42C	0.9800
C15—H15C	0.9800	C43—H43A	0.9800
C16—H16A	0.9800	C43—H43B	0.9800
C16—H16B	0.9800	C43—H43C	0.9800
C16—H16C	0.9800	C44—C45	1.513 (5)
C21—C22	1.517 (5)	C44—C46	1.520 (6)
C21—C23	1.517 (5)	C44—H44	1.0000
C21—H21	1.0000	C45—H45A	0.9800
C22—H22A	0.9800	C45—H45B	0.9800
C22—H22B	0.9800	C45—H45C	0.9800
C22—H22C	0.9800	C46—H46A	0.9800
C23—H23A	0.9800	C46—H46B	0.9800
C23—H23B	0.9800	C46—H46C	0.9800
C23—H23C	0.9800		
			
N1—O1—Be2	113.4 (2)	C26—C24—C25	110.5 (3)
O1—N1—C14	106.6 (2)	N2—C24—H24	107.0
O1—N1—C11	105.0 (2)	C26—C24—H24	107.0
C14—N1—C11	111.8 (3)	C25—C24—H24	107.0
O1—N1—Be1	107.4 (3)	C24—C25—H25A	109.5
C14—N1—Be1	110.8 (3)	C24—C25—H25B	109.5
C11—N1—Be1	114.6 (3)	H25A—C25—H25B	109.5
O4—Be1—O3	144.9 (3)	C24—C25—H25C	109.5
O4—Be1—N1	112.9 (3)	H25A—C25—H25C	109.5
O3—Be1—N1	102.2 (3)	H25B—C25—H25C	109.5
O4—Be1—Be2	179.4 (3)	C24—C26—H26A	109.5
O3—Be1—Be2	35.46 (16)	C24—C26—H26B	109.5
N1—Be1—Be2	66.72 (19)	H26A—C26—H26B	109.5
N2—O2—Be2	113.9 (3)	C24—C26—H26C	109.5
O2—N2—C24	106.3 (3)	H26A—C26—H26C	109.5
O2—N2—C21	105.3 (2)	H26B—C26—H26C	109.5
C24—N2—C21	112.7 (3)	N3—C31—C32	109.3 (3)
O2—Be2—O1	129.0 (3)	N3—C31—C33	116.6 (3)
O2—Be2—O3	124.3 (3)	C32—C31—C33	111.5 (4)
O1—Be2—O3	106.7 (3)	N3—C31—H31	106.2
O2—Be2—Be1	158.5 (3)	C32—C31—H31	106.2
O1—Be2—Be1	72.4 (2)	C33—C31—H31	106.2
O3—Be2—Be1	34.30 (16)	C31—C32—H32A	109.5
N3—O3—Be1	119.2 (2)	C31—C32—H32B	109.5
N3—O3—Be2	129.8 (3)	H32A—C32—H32B	109.5
Be1—O3—Be2	110.2 (3)	C31—C32—H32C	109.5
C34—N3—O3	107.3 (2)	H32A—C32—H32C	109.5
C34—N3—C31	114.6 (3)	H32B—C32—H32C	109.5
O3—N3—C31	107.2 (2)	C31—C33—H33A	109.5
N4—O4—Be1	126.9 (3)	C31—C33—H33B	109.5
O4—N4—C41	104.9 (2)	H33A—C33—H33B	109.5
O4—N4—C44	106.6 (3)	C31—C33—H33C	109.5
C41—N4—C44	112.3 (3)	H33A—C33—H33C	109.5
C13—C11—N1	112.1 (3)	H33B—C33—H33C	109.5
C13—C11—C12	110.4 (3)	N3—C34—C35	110.1 (3)
N1—C11—C12	108.7 (3)	N3—C34—C36	108.6 (3)
C13—C11—H11	108.5	C35—C34—C36	108.8 (4)
N1—C11—H11	108.5	N3—C34—H34	109.8
C12—C11—H11	108.5	C35—C34—H34	109.8
C11—C12—H12A	109.5	C36—C34—H34	109.8
C11—C12—H12B	109.5	C34—C35—H35A	109.5
H12A—C12—H12B	109.5	C34—C35—H35B	109.5
C11—C12—H12C	109.5	H35A—C35—H35B	109.5
H12A—C12—H12C	109.5	C34—C35—H35C	109.5
H12B—C12—H12C	109.5	H35A—C35—H35C	109.5
C11—C13—H13A	109.5	H35B—C35—H35C	109.5
C11—C13—H13B	109.5	C34—C36—H36A	109.5
H13A—C13—H13B	109.5	C34—C36—H36B	109.5
C11—C13—H13C	109.5	H36A—C36—H36B	109.5
H13A—C13—H13C	109.5	C34—C36—H36C	109.5
H13B—C13—H13C	109.5	H36A—C36—H36C	109.5
N1—C14—C16	113.9 (3)	H36B—C36—H36C	109.5
N1—C14—C15	109.4 (3)	N4—C41—C42	109.9 (3)
C16—C14—C15	110.3 (3)	N4—C41—C43	109.6 (3)
N1—C14—H14	107.7	C42—C41—C43	109.5 (3)
C16—C14—H14	107.7	N4—C41—H41	109.3
C15—C14—H14	107.7	C42—C41—H41	109.3
C14—C15—H15A	109.5	C43—C41—H41	109.3
C14—C15—H15B	109.5	C41—C42—H42A	109.5
H15A—C15—H15B	109.5	C41—C42—H42B	109.5
C14—C15—H15C	109.5	H42A—C42—H42B	109.5
H15A—C15—H15C	109.5	C41—C42—H42C	109.5
H15B—C15—H15C	109.5	H42A—C42—H42C	109.5
C14—C16—H16A	109.5	H42B—C42—H42C	109.5
C14—C16—H16B	109.5	C41—C43—H43A	109.5
H16A—C16—H16B	109.5	C41—C43—H43B	109.5
C14—C16—H16C	109.5	H43A—C43—H43B	109.5
H16A—C16—H16C	109.5	C41—C43—H43C	109.5
H16B—C16—H16C	109.5	H43A—C43—H43C	109.5
N2—C21—C22	110.1 (3)	H43B—C43—H43C	109.5
N2—C21—C23	109.0 (3)	N4—C44—C45	109.2 (3)
C22—C21—C23	109.3 (3)	N4—C44—C46	115.8 (3)
N2—C21—H21	109.4	C45—C44—C46	110.9 (3)
C22—C21—H21	109.4	N4—C44—H44	106.8
C23—C21—H21	109.4	C45—C44—H44	106.8
C21—C22—H22A	109.5	C46—C44—H44	106.8
C21—C22—H22B	109.5	C44—C45—H45A	109.5
H22A—C22—H22B	109.5	C44—C45—H45B	109.5
C21—C22—H22C	109.5	H45A—C45—H45B	109.5
H22A—C22—H22C	109.5	C44—C45—H45C	109.5
H22B—C22—H22C	109.5	H45A—C45—H45C	109.5
C21—C23—H23A	109.5	H45B—C45—H45C	109.5
C21—C23—H23B	109.5	C44—C46—H46A	109.5
H23A—C23—H23B	109.5	C44—C46—H46B	109.5
C21—C23—H23C	109.5	H46A—C46—H46B	109.5
H23A—C23—H23C	109.5	C44—C46—H46C	109.5
H23B—C23—H23C	109.5	H46A—C46—H46C	109.5
N2—C24—C26	115.6 (3)	H46B—C46—H46C	109.5
N2—C24—C25	109.3 (3)		

## References

[bb1] Berger, R. J. F., Hartmann, M., Pyykkö, P., Sundholm, D. & Schmidbaur, H. (2001). *Inorg. Chem.* **40**, 2270–2274. 10.1021/ic0007660 11327901

[bb2] Berger, R. J. F., Jana, S., Froehlich, R. & Mitzel, N. W. (2011). *Z. Naturforsch. Teil B*, **64**, 1131–1135.

[bb3] Berger, R. J. F., Schmidt, M. A., Jusélius, J., Sundholm, D., Sirsch, P. & Schmidbaur, H. (2001). *Z. Naturforsch. Teil B*, **56**, 979–989.

[bb4] Dressel, M. P., Nogai, S., Berger, R. J. F. & Schmidbaur, H. (2003). *Z. Naturforsch. Teil B*, **58**, 173–182.

[bb5] Neumüller, B. & Dehnicke, K. (2010). *Z. Anorg. Allg. Chem.* **636**, 515–517.

[bb6] Sheldrick, G. M. (2008). *Acta Cryst.* A**64**, 112–122. 10.1107/S0108767307043930 18156677

[bb7] Spek, A. L. (2009). *Acta Cryst.* D**65**, 148–155. 10.1107/S090744490804362X PMC2631630 19171970

[bb8] Stoe & Cie (2012). *X-AREA* and *X-RED32* Stoe & Cie, Darmstadt, Germany.

[bb9] Ullrich, M. (2007). PhD thesis, Universität Münster, Germany.

[bb10] Westrip, S. P. (2010). *J. Appl. Cryst.* **43**, 920–925.

